# Resting Heart Rate Variability Is Associated With Subsequent Orthostatic Hypotension: Comparison Between Healthy Older People and Patients With Rapid Eye Movement Sleep Behavior Disorder

**DOI:** 10.3389/fneur.2020.567984

**Published:** 2020-11-23

**Authors:** Yukiyoshi Sumi, Chikao Nakayama, Hiroshi Kadotani, Masahiro Matsuo, Yuji Ozeki, Takafumi Kinoshita, Yuki Goto, Manabu Kano, Toshitaka Yamakawa, Masako Hasegawa-Ohira, Keiko Ogawa, Koichi Fujiwara

**Affiliations:** ^1^Department of Psychiatry, Shiga University of Medical Science, Otsu, Japan; ^2^Department of Systems Science, Kyoto University, Kyoto, Japan; ^3^Department of Sleep and Behavioral Sciences, Shiga University of Medical Science, Otsu, Japan; ^4^Department of Priority Organization for Innovation and Excellence, Kumamoto University, Kumamoto, Japan; ^5^Faculty of Education, Shiga University, Otsu, Japan; ^6^Graduate School of Humanities and Social Sciences, Hiroshima University, Higashihiroshima, Japan; ^7^Department of Materials Process Engineering, Nagoya University, Nagoya, Japan

**Keywords:** orthostatic hypotension, OH, rapid eye movement sleep behavior disorder, RBD, heart rate variability, HRV, Poincaré plot, autonomic dysfunction

## Abstract

**Background:** Orthostatic hypotension (OH) caused by autonomic dysfunction is a common symptom in older people and patients with idiopathic rapid eye movement sleep behavior disorder (iRBD). The orthostatic challenge test is a standard autonomic function test that measures a decrease of blood pressure during a postural change from supine to standing positions. Although previous studies have reported that changes in heart rate variability (HRV) are associated with autonomic dysfunction, no study has investigated the relationship between HRV before standing and the occurrence of OH in an orthostatic challenge test. This study aims to examine the connection between HRV in the supine position and the occurrence of OH in an orthostatic challenge test.

**Methods:** We measured the electrocardiograms of patients with iRBD and healthy older people during an orthostatic challenge test, in which the supine and standing positions were held for 15 min, respectively. The subjects were divided into three groups: healthy controls (HC), OH-negative iRBD [OH (–) iRBD], and OH-positive iRBD [OH (+) iRBD]. HRV measured in the supine position during the test were calculated by time-domain analysis and Poincaré plots to evaluate the autonomic dysfunction.

**Results:** Forty-two HC, 12 OH (–) iRBD, and nine OH (+) iRBD subjects were included. HRV indices in the OH (–) and the OH (+) iRBD groups were significantly smaller than those in the HC group. The multivariate logistic regression analysis for OH identification for the iRBD groups showed the model whose inputs were the HRV indices, i.e., standard deviation 2 (SD2) and the percentage of adjacent intervals that varied by more than 50 ms (pNN50), had a receiver operating characteristic curve with area under the curve of 0.840, the sensitivity to OH (+) of 1.000, and the specificity to OH (–) of 0.583 (*p* = 0.023).

**Conclusions:** This study showed the possibility that short-term HRV indices in the supine position would predict subsequent OH in iRBD patients. Our results are of clinical importance in terms of showing the possibility that OH can be predicted using only HRV in the supine position without an orthostatic challenge test, which would improve the efficiency and safety of testing.

## Introduction

Orthostatic hypotension (OH) is a major health concern in older people, affecting approximately one-third to two-thirds of them (1–3). A recent systematic review and meta-analysis found that OH is associated with falls in older people (4). OH symptoms, which include dizziness, titubation, blurry vision, syncope, nausea, and falls, occur due to temporary cerebral hypoperfusion and sympathetic hyperactivity (5). In particular, it is important to prevent falls associated with OH because injuries caused by falls may significantly impair quality of life.

OH is defined as a persistent decrease of systolic blood pressure (BP) ≥20 mm Hg, a persistent decrease of diastolic BP ≥10 mm Hg, or a decrease in systolic BP to <90 mm Hg within 3 min of standing or head-up tilt, or the manifestation of any OH clinical symptoms (2, 6, 7). It has been reported that a drop in BP at 1 min after standing from supine is important because it is associated with future adverse outcomes, including falls, fractures, and syncope (8). The American Society of Hypertension recommends measuring BP at 1 and 3 min after standing to detect BP decrease within 3 min (9). While aging, drug side effects, and dehydration can cause OH, autonomic dysfunction is one of the most important factors of OH, but it is often difficult to be aware of it (5, 10).

Rapid eye movement (REM) sleep behavior disorder (RBD) is REM parasomnia characterized by dream-enacting behaviors (11). Although RBD is widespread among patients with neurodegenerative disorders, such as Parkinson's disease (PD), dementia with Lewy bodies (DLB), or multiple system atrophy (MSA) (12–17), RBD is often a precursor of the clinical appearance of PD, DLB, or MSA. The idiopathic form without any neurological disorder is called idiopathic RBD (iRBD) (18). Although some iRBD patients have stayed disease-free for more than 10 years after being diagnosed with RBD (19), most iRBD patients phenoconvert to synucleinopathy over time (20), and it has been reported that severe cardiovascular autonomic dysfunction in iRBD is associated with phenoconversion to DLB (21).

In order to prevent falls of iRBD patients caused by OH, the severity of OH needs to be evaluated. One standard test for evaluating the severity of OH is the orthostatic challenge test, which usually takes place in clinics or hospitals (3). In the test, a subject is asked to change his/her position from supine to standing, and his/her BP is measured at fixed intervals during both the supine and the standing positions. Since the orthostatic challenge test requires medical staff to ensure subject safety, it is difficult to perform it in daily life. Thus, we should develop a simple alternative method for evaluating OH severity that can be easily performed even in daily life.

Autonomic dysfunction and OH are common among older people, particularly patients with α-synucleinopathies, such as PD, DLB, and MSA (10, 22). It has been reported that the more severe motor symptoms become, the more severe the autonomic dysfunction becomes in patients with PD (23).

The pathology of RBD involves brain regions that are responsible for the autonomic nervous system as well as the brainstem which regulates REM sleep (24, 25). Various clinical studies have reported that autonomic dysfunction is associated with iRBD. According to a multicentral study involving 24 institutes, 156 out of 531 patients with iRBD (29.4%) had orthostatic symptoms (26). In addition, the uptake of ^123^I-metaiodobenzylguanidine (^123^I-MIBG) in the iRBD patients tends to decrease, which indicates cardiac sympathetic denervation (27, 28). Thus, patients with iRBD potentially have autonomic dysfunction, and the severity of autonomic dysfunction may be utilized as a proxy variable for assessing OH severity.

Although various methods for evaluating the severity of autonomic dysfunction have been proposed, they have some problems. Interviews or questionnaires such as the Scales for Outcomes in Parkinson's Disease-Autonomic questionnaire (SCOPA-AUT) (23) may overlook OH symptoms of which patients are not aware. ^123^I-MIBG is a quantitative test of autonomic dysfunction; however, it uses a radioactive substance and is expensive.

In this work, we focus on heart rate variability (HRV) which is spontaneous beat-to-beat oscillation in the R-R interval (RRI) of an electrocardiogram (ECG) and reflects the autonomic nervous function (29). According to research using RRI data collected during sleep by means of polysomnography (PSG), HRV indicators of patients with PD and iRBD are different from healthy controls (30–32), which indicates that patients with PD and iRBD have autonomic dysfunction. However, studies on HRV during an OH test of iRBD patients have not been performed.

We assumed that signs of autonomic dysfunction associated with OH appear even when in the supine position before standing in the orthostatic challenge test. Therefore, we conducted the orthostatic challenge test with ECG measurement for patients with iRBD and healthy older people in order to investigate the relationship between HRV in the supine position and the occurrence of OH. In addition, we discuss the possibility of the use of HRV analysis as an alternative method to the orthostatic challenge test for evaluating OH severity.

## Methods

### Subjects

Participants in this study consisted of healthy subjects and patients with iRBD. The details of this study were explained to them and written informed consent was obtained from each subject. In addition, the protocol was approved by the ethical committee at the Shiga University of Medical Science (R2017-199). Healthy subjects and patients with iRBD were recruited between December 2017 and August 2019.

We recruited healthy subjects over 60 years of age and without history of autonomic dysfunction.

In this study, patients who satisfied diagnostic criteria of RBD according to the 3rd edition of the International Classification of Sleep Disorders (ICSD-3) were recruited from among patients who visited the Shiga University of Medical Science. PSG was performed on all of the patients; who were then diagnosed with RBD. In order to focus on iRBD (18), patients with PD (33), DLB (34), or MSA (35) were excluded. Patients on antidepressants or with severe sleep apnea were also excluded because they might have symptoms mimicking RBD, such as medication-related dream enactment behaviors and apnea-related behaviors (36, 37). Patients with a history of cerebral infarction were also excluded.

**Figure 1:**
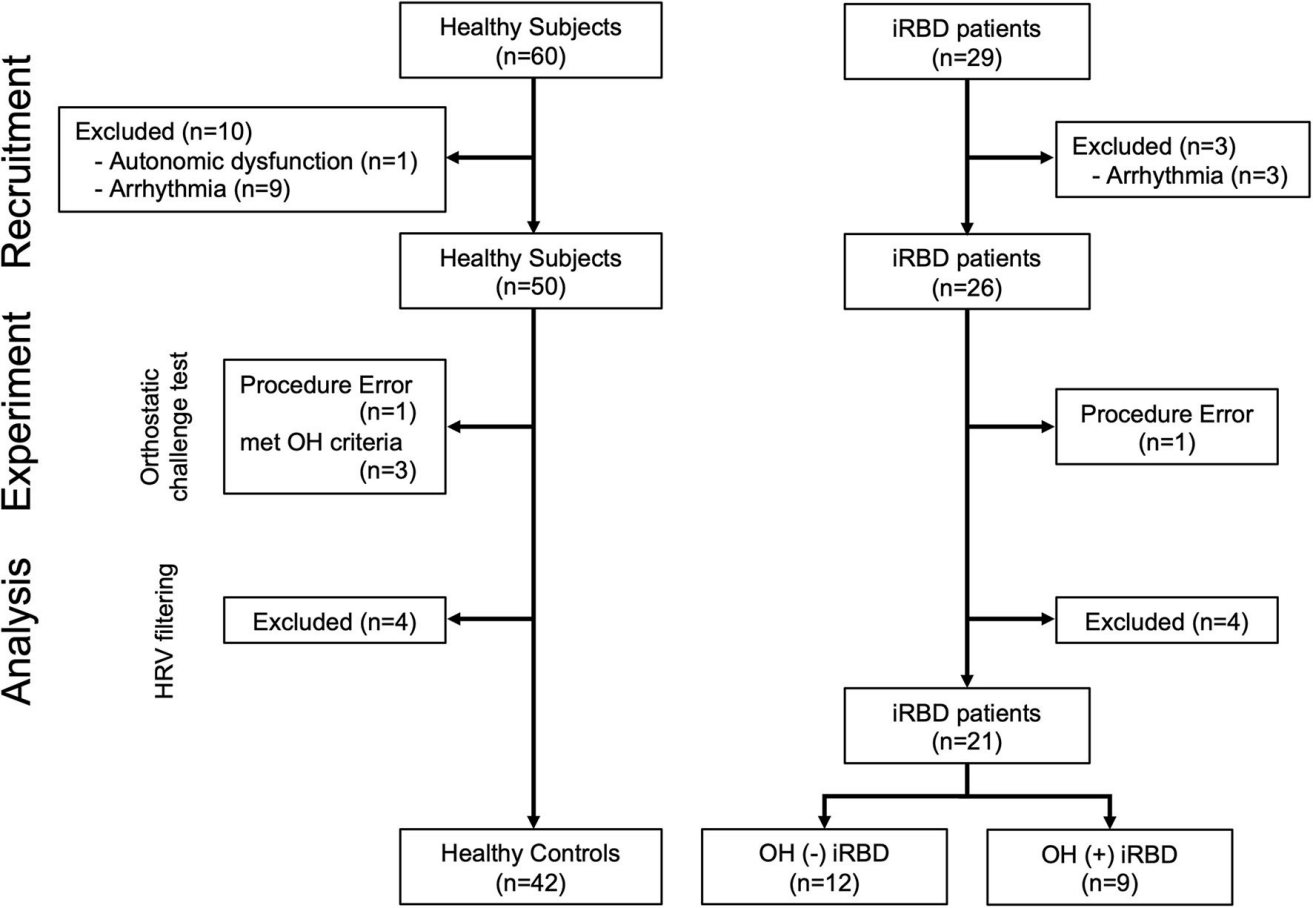
Flowchart of subject enrollment. iRBD, idiopathic rapid eye movement sleep behavior disorder; OH, orthostatic hypotension; HRV, heart rate variability.

A total of 60 healthy subjects and 29 iRBD patients were enrolled. One healthy subject was excluded because of a history of autonomic failure. Since a comorbidity of arrhythmia could affect the results of HRV analysis, 9 healthy participants and 3 iRBD patients with the comorbidity of arrhythmia were excluded (Figure 1).

The orthostatic challenge test (the details are described in the “Experimental Protocol” section) was conducted on the remaining 50 healthy subjects and 26 iRBD patients. One healthy subject who did not maintain the standing position during the standing phase was excluded, and 3 healthy subjects who satisfied the OH criteria were also excluded (the details of OH criteria are described in “Orthostatic Hypotension Criteria and Grouping” in the “Orthostatic Challenge Test” section). One iRBD patient to whom the examiner failed to attach devices was also excluded. The adaptation of the Hampel and the quotient filters (38, 39) resulted in the exclusion of 4 healthy subjects and 4 patients with iRBD.

Finally, 42 healthy controls (HC) and 21 patients with iRBD [12 OH (–) iRBD and 9 OH (+) iRBD] were included in this study. The definition of the subject grouping are described in “Orthostatic Hypotension Criteria and Grouping” in the “Orthostatic Challenge Test” section.

### Experimental Protocol

The study consisted of a clinical interview and an orthostatic challenge test. The participants were instructed not to ingest alcohol, caffeine, or to smoke the night before the experiment because these substances could affect the autonomic nervous system (40). The experiment was conducted between 2 p.m. and 4 p.m. to minimize the effect of the circadian rhythm on HRV (41). Subjects were instructed to finish meals 2 h before the start of the experiment (42).

#### Clinical Interview

The subjects were interviewed regarding their past histories including arterial hypertension, coronary artery disease, myocardial infarction, and diabetes mellitus, as well as their smoking habits, which could affect BP dynamics and autonomic nervous functions (43). Interviews about medication which could affect BP or HRV, including beta-blockers, calcium channel blockers, diuretics, angiotensin-converting-enzyme (ACE) and angiotensin II type 1 (AT II) inhibitors, organic nitrates, arrhythmic medications, atropine, scopolamine, atenolol, metoprolol, loop diuretics, monoamine oxidase inhibitors, antipsychotics, antidepressant agents, phenothiazine, phosphodiesterase type 5 inhibitors, parkinsonism agents, barbiturates, anesthetics, opioids, muscle relaxants, vincristine, or doxorubicin were also conducted (7, 43–49). The cognitive functions of the subjects were evaluated using the Mini-Mental State Examination (MMSE), which is a screening tool for dementia with a cut-off score of 23/24 (maximum score of 30) (50). The participants were asked to answer the REM sleep behavior disorder screening questionnaire (RBDSQ), which consists of 13 questions (maximum possible score = 13), to assess RBD symptoms such as nocturnal movements, injuries, and motor behavior during the night (51, 52). In this study, the Japanese version of the RBDSQ (RBDSQ-J) was used (53).

In addition, patients with iRBD were asked about the duration of their RBD disease, the onset of which was confirmed by family members.

#### Orthostatic Challenge Test

In order to evaluate behavior in daily life, an active standing test called the orthostatic challenge test was performed instead of passive standing tests such as the tilt test (3). The orthostatic challenge test was performed in our laboratories, in which the temperature and humidity were set to 23.0 ± 2.0°C [mean ± standard deviation (SD)] and 35.2 ± 12.7 % (mean ± SD), respectively.

##### Measurement Devices

**Figure 2:**
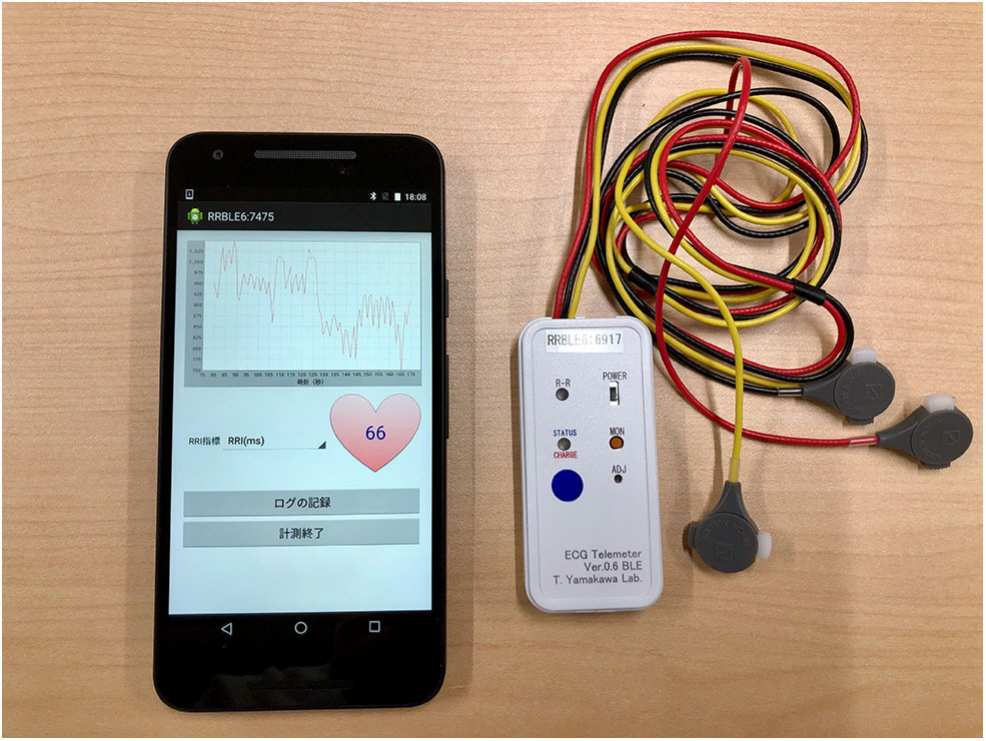
Smartphone Nexus 5X and wearable RR intervals sensor. The RRI data acquired by the wearable RRI sensor was sent to the smartphone application in real time. This figure shows the heart rate 66 calculated from the RRI at that time. In this experiment, the electrodes of the wearable RRI sensor were attached to the three poles of the trunk of the subject, just as in a normal electrocardiogram. The smartphone was in a running pouch attached to the back of the waist of the subject. RRI, RR intervals.

A wearable RRI sensor (T. Yamakawa Lab, wireless R-R monitor Bluetooth LE model ver 0.7) was used for RRI measurement (Figure 2) (54). This sensor is able to record ECG signals at a 1,000 Hz sampling rate and to measure RRI automatically without requiring any special skills. The measured RRI data were sent to a Nexus5X smartphone [Google, LG. Operating system Android 6.0 (Marshmallow)] via Bluetooth Low Energy (BLE). The smartphone was placed in a running pouch attached to the back of the waist and the received RRI data were stored by a custom-made smartphone app (Figure 2) (55). The RRI error handling procedure in this study is described in the “Heart Rate Variability Analysis” section.

In addition, BP and pulse rate (PR) were measured with a digital brachial BP monitor HEM-7500 (OMRON HEALTHCARE Co., Ltd., Japan). The BP monitor was attached to the left upper arm of the participant so that the cuff of the sphygmomanometer was at heart level. When the examiner pressed a button on the sphygmomanometer, the cuff was automatically pressurized and depressurized to record systolic BP (sBP), diastolic BP (dBP), and PR.

##### Orthostatic Challenge Protocol

**Figure 3:**
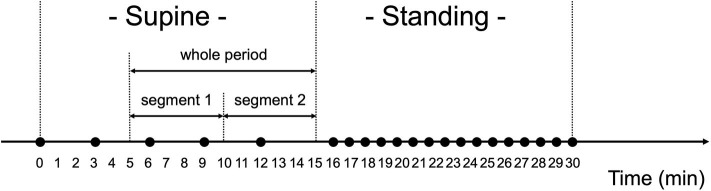
Orthostatic challenge protocol. Black circles (•) show when blood pressure and pulse rate were measured. The measurements were done every 3 min when in the supine position, and every 1 min when in the standing position. Ten min worth of RRI data in the supine position were analyzed for heart rate variability, which were divided into two segments (segment 1: 6–10 min and segment 2: 11–15 min).

The orthostatic challenge test was composed of two phases: supine- and standing-position phases (Figure 3). Before the test, the participants were equipped with the wearable RRI sensor, the smartphone, and the sphygmomanometer, and instructed to lie in bed at about 40 cm from the floor. In addition, they were instructed to avoid talking and not to fall asleep during the test.

After checking the device operation in the supine position (>2 min), the measurements of BP and PR began, they were performed four times with 3-min intervals. After being in the supine position for 15 min, the participants were instructed to quickly stand up and to keep their standing position for 15 min. After standing for 1 min, BP and PR measurement was performed once per min for 15 min. The examiner carefully monitored the safety of the participant during the test. After the orthostatic challenge test, the examiner, by asking the participants, confirmed the presence/absence of the following symptoms: dizziness, titubation, blurry vision, syncope, or nausea.

##### Orthostatic Hypotension Criteria and Grouping

The average of five BP and PR measurements of each participant in the supine position was defined as the baseline. The orthostatic challenge test was determined to be positive when the participant satisfied one of the following conditions: (1) decrease of sBP ≥20 mm Hg, or decrease of dBP ≥10 mm Hg in comparison with the baseline BP at 1 or 3 min after standing, (2) a decrease in systolic BP to <90 mm Hg, or (3) manifestation of any clinical symptom including falling, dizziness, blurry vision, syncope, and nausea (2, 6, 7, 9). However, for the patients with baseline sBP ≥160 mm Hg, the criterion of a decrease of sBP ≥30 mm Hg was applied thereto, based on criteria proposed by the American Society of Hypertension (3, 9). In all other cases, the orthostatic challenge test was determined to be negative.

In patients with iRBD, those who met the orthostatic challenge test criteria were defined as “OH (+) iRBD,” and those who did not meet the criteria were defined as “OH (–) iRBD.” Among healthy subjects, those who did not meet the OH criteria were defined as “HC (healthy control);” on the other hand, those who met the OH criteria were excluded because they might have potential autonomic nervous dysfunction (Figure 1).

### Heart Rate Variability Analysis

The HRV data extracted from the RRI data measured when in the supine position were analyzed (Figure 3). Data from the first 5 min in the supine position were excluded from the HRV analysis because RRI for the first 5 min may have been impacted by the posture change from the sitting to the supine position (56). Thus, data from the remaining 10 min were analyzed, which were named “whole period” and divided into two segments (segment 1: 6–10 min and segment 2: 11–15 min). In the manuscript, the results for segment 2 were mainly described because the subjects had been in the supine position for a long time; the results for segment 1 and the whole period are shown in the Supplementary Table 1.

Incidental arrhythmias such as premature atrial contractions (PAC) or premature ventricular contractions (PVC) are often observed in continuous ECG measurements of elderly people, although arrhythmias are not identified in short-term ECG examination. A high proportion of PAC and PVC in the elderly has been reported (57, 58); thus, it is necessary to filter ectopic RRI caused by PAC or PVC to suppress the influence of such ectopic RRIs on the HRV analysis (59). Therefore, this study adopted two HRV filtering methods: the Hampel filter and the quotient filter (38, 39). RRIs that deviate from the 3-σ range, which refers to data within three standard deviations from a mean, are detected by means of the Hampel filtering. In addition, quotient filtering detects significant RRI fluctuations: RRI that has a 10% or more change than the mean of before and after the RRI. Additionally, all data recorded from the participant were removed from the analysis when the sum of the RRIs detected with these filters became >150 s within the 5-min section (or 300 s within the 10-min section), because reliable analysis of such ectopic data was difficult.

**Figure 4:**
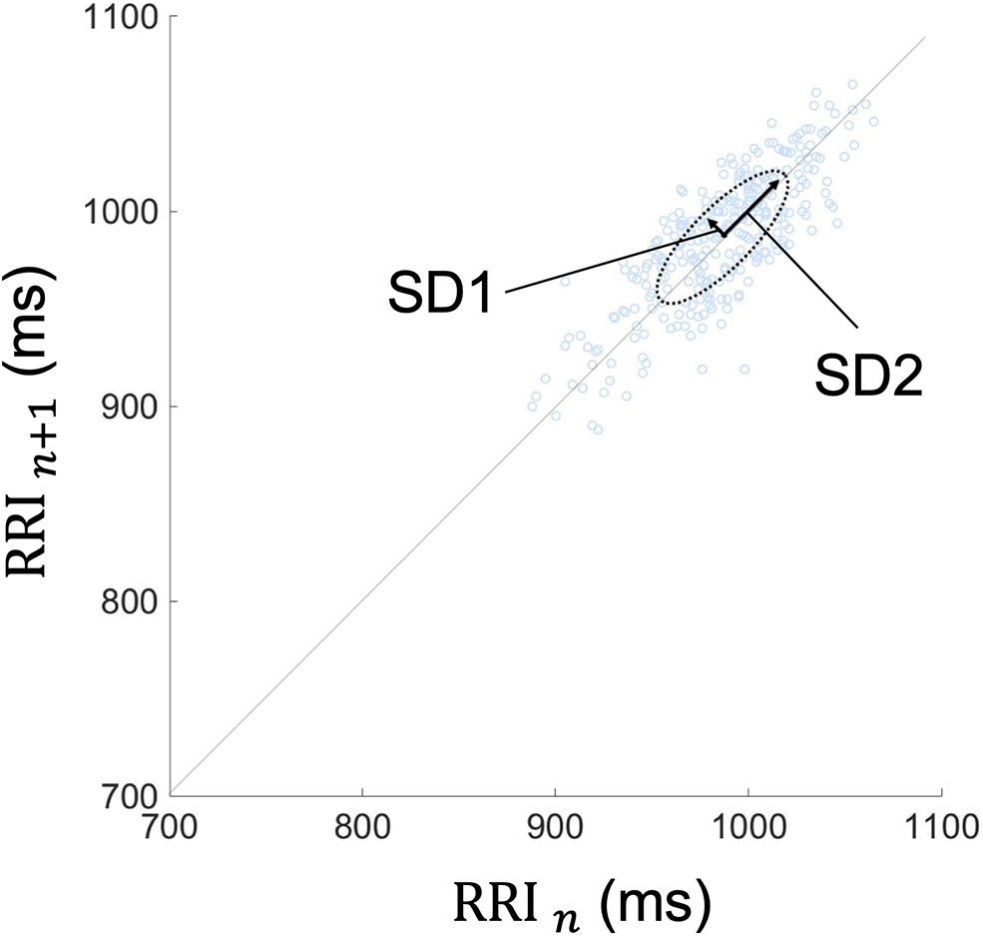
Typical Poincaré plot of RRI. The Poincaré plot was based on a simple scatter of plots where the x value is *n*th RRI and its corresponding y value is the (*n* + 1)th RRI. Each plot is described in the (RRI *n*, RRI *n* + 1) space. This figure represents the Poincaré plot of a 5-min RRI. The Poincaré plot typically forms an ellipse with about a 45-degree tilt, and the diffusion range around the major axis and the minor axis of the ellipse are defined as SD2 and SD1, respectively. Their ratio is defined as SD1/SD2. RRI, RR intervals; SD1, standard deviation 1; SD2, standard deviation 2.

Because frequency-domain analysis is significantly affected by incidental arrhythmias, the Poincaré plot and time-domain analysis were employed in this study (60). The Poincaré plot is based on a simple scatter of plots where the x value is the *n*th RRI and its corresponding y value is the (*n* + 1)th RRI. A typical Poincaré plot forms an ellipse with about a 45 tilt, and the diffusion ranges around the major axis and the minor axis of the ellipse are defined as the standard deviation 2 and 1 (SD2 and SD1), respectively. In addition, their ratio is defined as SD1/SD2 (Figure 4).

In addition to the Poincaré plot, time-domain analysis, which includes the standard deviation of all R-R intervals (SDNN), the root mean square of successive differences (RMSSD), and the percentage of adjacent intervals that varied by more than 50 ms (pNN50) was performed.

Various indices of HRV have been reported to be associated with the sympathetic and the parasympathetic nervous system (61). For the time-domain analysis, SDNN is influenced by the sympathetic and the parasympathetic nervous system; pNN50 and RMSSD mainly correlate to the parasympathetic nervous system. In particular, pNN50 <3% and RMSSD <25 ms are regarded as low parasympathetic activity (62, 63). For the Poincaré plots, SD1 represents the parasympathetic nervous function, SD2 is related more strongly to the sympathetic than to the parasympathetic tone, and SD1/SD2 represents the balance between the sympathetic-parasympathetic arms (60, 64–66).

### Outcome Measurement

The demographic data of the HC, OH (–) iRBD, and OH (+) iRBD groups were examined. In addition, the experimental data of these three groups—BP and PR of the baseline and the delta values at 1 and 3 min after standing, and the ratio of clinical symptoms manifested after standing—were compared. The HRV features when in the supine position were compared among the three groups.

### Statistical Analysis

For comparison with the three groups, nominal data were compared using the χ^2^-test. The normality of the distribution of the variable was checked by the Shapiro-Wilk test (*p* < 0.05 was considered statistically significant). Parametric variables were presented as mean ± SD; non-parametric variables were presented as the median and interquartile range (IQR). For the three groups, parametric variables were compared using one-way analyses of variance (ANOVAs) with Tukey's *post-hoc* analysis followed by Bonferroni correction; non-parametric variables were compared using the Kruskal-Wallis test and *post-hoc* Mann-Whitney test followed by Bonferroni correction. For comparison with the two groups [OH (–) iRBD and OH (+) iRBD], parametric variables were compared using Student's *t*-test.

Age and BMI were compared using ANOVAs. Years of education, a score of MMSE, and a score of RBDSQ-J were compared using the Kruskal-Wallis test. Gender differences, rate of comorbidities, and rate of medication usage were compared using the χ^2^-test. The disease duration of RBD was analyzed using the Student's *t*-test. In the orthostatic challenge test, BP and PR of the baseline and the delta values at 1 and 3 min after standing were compared using ANOVAs at each time point. For the correction for the multiple comparisons, Tukey's *post-hoc* analysis was used for the comparison of multiple groups, and Bonferroni adjustment was performed for multiple time points (*p* < 0.05/3 ≈ 0.017). The ratio of the clinical symptoms that appeared after standing was assessed using the χ^2^-test. In addition, HRV indices when in the supine position were compared using the Kruskal-Wallis test and *post-hoc* Mann-Whitney test followed by Bonferroni correction.

A value of *p* < 0.05 was considered significant in this study. For parametric variables, the Cohen's *d* effect size index was used to calculate the pairwise differences. Cohen classified the effect sizes into small (*d* = 0.2), medium (*d* = 0.5), and large (*d* = 0.8) (67). For non-parametric variables, the effect size *r* was used to calculate the pairwise differences. The effect sizes of *r* were classified into small (*r* = 0.1), medium (*r* = 0.3), and large (*r* = 0.5) (68).

Next, among the OH (–) iRBD and OH (+) iRBD patients, a multivariate logistic regression analysis was conducted to focus on the relationship between the OH (+/–) and HRV indices, with OH (–) or OH (+) as the dependent variable and HRV indices (SDNN, RMSSD, pNN50, SD1, SD2, and SD1/SD2) as the independent variables. The Bayesian information criterion (BIC) was used to select the appropriate independent variables. In a model that minimized the BIC, we calculated the area under the curve (AUC) of the receiver operating characteristic (ROC) curve, the sensitivity of OH (+), and the specificity of OH (–). Besides, the value of HRV indices that maximizes Youden's index was calculated to determine the optimal cutoff values (69).

These statistical tests were performed using IBM SPSS Statistics for Macintosh, Version 22.0 (IBM Corp. Armonk, NY). HRV were evaluated with an HRV Analysis software (Kubios HRV v.1.1 for Windows, Biomedical Signal Analysis Standard).

## Results

### Demographics

The demographic data of the included participants are shown in Table 1. HC, OH (–) iRBD, and OH (+) iRBD subjects were not significantly different in age, BMI, years of education, or the scores of MMSE. Since the patients were predominantly male, reflecting the male-dominant nature of this disease, the sex proportions were significantly different among the three groups. The RBDSQ-J scores were significantly different among the three groups. The RBD disease durations were not significantly different between OH (–) and OH (+) iRBD groups.

**Table 1:** Demographic data of healthy controls, OH (–) iRBD, and OH (+) iRBD groups.

**Demographics**	**HC** **(*n* = 42)**	**OH (–) iRBD** **(*n* = 12)**	**OH (+) iRBD** **(*n* = 9)**	***p***
Age (years)^a^	70.2 ±6.6	74.8 ±6.3	73.2 ±5.3	0.068
Gender (male: female)^b^	16:26	9:3	8:1	**0.005**
BMI (kg/m^2^)^a^	22.4 ±3.0	21.6 ±2.6	23.2 ±2.9	0.468
Years of education^c^	13.0 (4.0)	16.0 (4.0)	12.0 (4.0)	0.347
MMSE^c^	29.0 (3.0)	29.0 (4.0)	29.0 (5.0)	0.958
RBDSQ-J^c^	1.0 (2.0)	3.0 (2.5)	3.0 (3.0)	**0.002**
Duration of RBD (years)^d^	–	10.2 ±6.8	9.2 ±6.2	0.748
Comorbidities^b^				
Arterial hypertension (*n*)	9 (21.4%)	4 (33.3%)	3 (33.3%)	0.592
Coronary artery disease (*n*)	1 (2.4%)	2 (16.7%)	1 (11.1%)	0.165
Myocardial infarction (*n*)	0 (0.0%)	0 (0.0%)	2 (22.2%)	**0.002**
Diabetes mellitus (*n*)	3 (7.1%)	3 (25.0%)	1 (11.1%)	0.222
Medication^b^				
Calcium channel blockers (*n*)	6 (14.3%)	3 (25.0%)	3 (33.3%)	0.353
ACE and AT II inhibitors (*n*)	4 (9.5%)	1 (8.3%)	2 (22.2%)	**<0.001**
Organic nitrates (*n*)	1 (2.4%)	1 (8.3%)	0 (0.0%)	0.492

*ACE, angiotensin-converting-enzyme; AT II, angiotensin II type 1; BMI, body mass index; HC, healthy control; OH, orthostatic hypotension; iRBD, idiopathic rapid eye movement sleep behavior disorder; MMSE, Mini-Mental State Examination; RBDSQ-J, rapid eye movement sleep behavior disorder screening questionnaire Japanese version*.

*Results are presented with mean ± standard deviation for parametric variables, median (interquartile range), or percentage in parentheses*.

*p < 0.05 was considered significant. Significant values are given in bold*.

a *One-way ANOVA*.

b *Chi-square test*.

c *Kruskal-Wallis test*.

d *Student t-test*.

Of the comorbidities that could affect BP or HRV, there were no significant differences in the promotions of arterial hypertension, coronary artery disease, and diabetes mellitus. However, the proportion of myocardial infarction was significantly higher in the OH (+) iRBD group.

For medications that could affect BP or HRV, calcium channel blockers, ACE and AT II inhibitors, and organic nitrates were used by the participants. There was a significant difference in the proportion of ACE and AT II inhibitor use. Of the calcium channel blockers, diltiazem may decrease the low-frequency component of HRV and nifedipine may not (45). ACE and AT II inhibitors may increase the low-frequency component of HRV (44). Nobody used any of the other medications listed below, which might influence BP or HRV; arrhythmic medications, atropine, scopolamine, atenolol, metoprolol, beta-blockers, loop diuretics, monoamine oxidase inhibitors, antipsychotics, antidepressant agents, phenothiazine, phosphodiesterase type 5 inhibitors, parkinsonism agents, barbiturates, anesthetics, opioids, muscle relaxants, vincristine, or doxorubicin.

As to smoking habit and the use of dopaminergic agonists, there were few participants (zero or one) in each group of HC, OH (–), and OH (+) iRBD.

### Orthostatic Challenge

**Figure 5:**
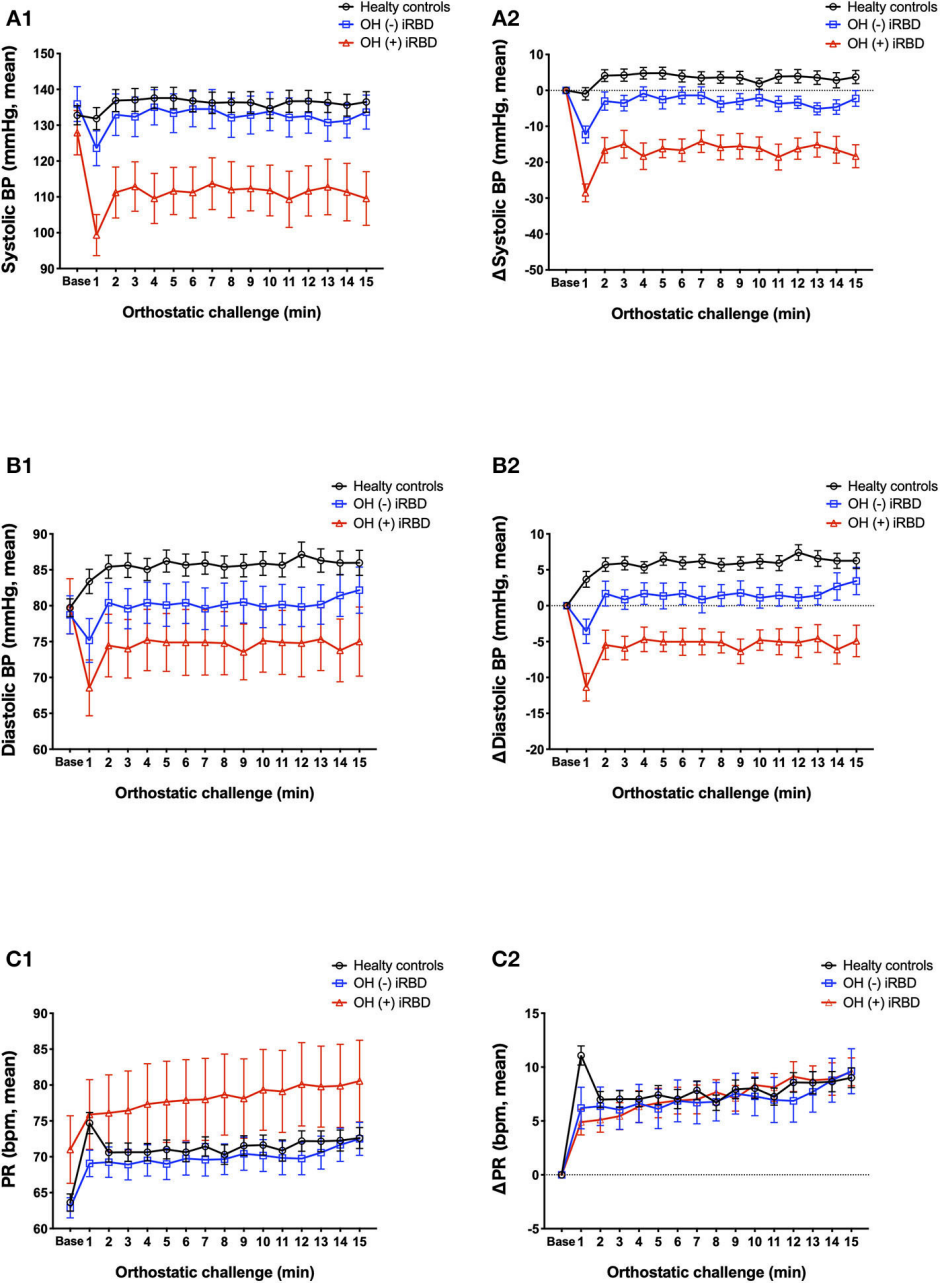
Comparison of orthostatic challenge test among healthy controls, OH (–) iRBD, and OH (+) iRBD groups. **(A1–C1)**, absolute changes (mean ± standard error of the mean); **(A2–C2)**, delta values compared to baseline supine position (mean ± standard error of the mean). BP, blood pressure; HR, heart rate; iRBD, idiopathic rapid eye movement sleep behavior disorder; OH, orthostatic hypotension. x-axis: base, mean baseline values in supine position; 1–15 min, after standing.

The results of the orthostatic challenge test are shown in Figure 5 and Table 2. sBP tended to decrease after standing, in the order of OH (+) iRBD, OH (–) iRBD, and HC (Figures 5A1,A2). In the OH (+) iRBD group, sBP decreased remarkably by 28.6 mm Hg on average at 1 min after standing, and the trend of decreasing sBP continued thereafter. dBP increased after standing in HC; however, it decreased in OH (+) iRBD (Figures 5B1,B2). In addition, dBP decreased by 11.4 mm Hg on average at 1 min after standing and the trend of decreasing dBP continued thereafter as with sBP.

**Table 2:** Results of orthostatic challenge test.

						**Effect sizes (Cohen's** ***d*****)**		
	**HC**	**OH (–) iRBD**	**OH (+) iRBD**			**HC–**	**HC–**	**OH (–) iRBD–**
	**(*n* = 42)**	**(*n* = 12)**	**(*n* = 9)**	***p***	**Pairwise differences**	**OH (–) iRBD**	**OH (+) iRBD**	**OH (+) iRBD**
**Systolic BP (mm Hg)^a^**								
Baseline	132.8 ±17.3	135.9 ±16.8	127.9 ±18.5	0.580	N/A	−0.181	0.280	0.456
ΔsBP at 1 min after standing	−1.0 ±11.2	−12.3 ±8.3	−28.6 ±7.5	**<0.001**	HC > OH (–) iRBD > OH (+) iRBD	**1.068**	**2.585**	**2.037**
ΔsBP at 3 min after standing	4.3 ±11.1	−3.6 ±7.5	−15.0 ±11.6	**<0.001**	HC > OH (+) iRBD	0.752	**1.727**	1.215
**Diastolic BP (mm Hg)^a^**								
Baseline	79.7 ±8.0	78.7 ±9.2	79.9 ±11.6	0.936	N/A	0.118	−0.023	−0.115
ΔdBP at 1 min after standing	3.7 ±7.1	−3.6 ±5.9	−11.4 ±5.8	**<0.001**	HC > OH (–) iRBD = OH (+) iRBD	**1.057**	**2.182**	1.334
ΔdBP at 3 min after standing	5.9 ±6.0	0.9 ±4.7	−5.9 ±4.9	**<0.001**	HC > OH (+) iRBD	0.879	**2.022**	1.411
**Pulse rate (beats/min)^a^**								
Baseline	63.6 ±7.8	62.9 ±7.8	71.0 ±14.1	0.055	N/A	0.100	−0.805	−0.819
ΔPR at 1 min after standing	11.1 ±5.7	6.2 ±6.7	4.9 ±3.6	**0.003**	HC > OH (+) iRBD	0.818	**1.135**	0.234
ΔPR at 3 min after standing	7.0 ±5.1	6.0 ±6.3	5.4 ±3.8	0.646	N/A	0.186	0.324	0.110
**Subjective symptoms^b^**								
Dizziness	0 (0.0%)	0 (0.0%)	2 (22.2%)	**0.002**				

*OH, orthostatic hypotension; iRBD, idiopathic rapid eye movement sleep behavior disorder; BP, blood pressure; PR, pulse rate*.

*Results are presented with mean ± standard deviation or percentage in parentheses*.

*One-way analysis of variance (ANOVA) for systolic BP, diastolic BP and PR, or Kruskal-Wallis for nominal variables are conducted*.

*p < 0.05 was considered significant. Significant values are given in bold*.

*Effect sizes (Cohen's d) are shown for pairwise difference*.

a *One-way ANOVA and Tukey's post-hoc analysis with Bonferroni correction for multiple comparison of three time points*.

b *Chi-square test*.

PR increased after standing in all three groups (Figures 5C1,C2). Although the increase in PR at 1 min after standing was 11.1 beats/min on average in HC, the increase of PR in OH (+) iRBD was 4.9 beats/min and smaller than that of HC (*p* = 0.003). PR in the HC group increased greatly after standing and then decreased rapidly. Similar changes were not observed in the OH (+) or OH (–) iRBD groups.

Delta values of BP and PR (written as ΔsBP, ΔdBP, and ΔPR) and the occurrence of subjective symptoms related to the OH +/– criteria were compared among the three groups (Table 2). sBP, dBP, and PR at the baseline were not significantly different among the three groups. There were statistically significant differences in ΔsBP at 1 min (*p* < 0.001) and at 3 min after standing (*p* < 0.001), and ΔdBP at 1 min (*p* < 0.001) and at 3 min after standing (*p* < 0.001). Subsequent *post-hoc* analyses revealed that ΔsBP at 1 min after standing in the OH (+) iRBD group was significantly lower than that in the HC group and OH (–) iRBD group with large effect sizes (Cohen's *d* = 2.585, 2.037, respectively). Similarly, ΔsBP at 3 min after standing in the OH (+) iRBD group was significantly lower than the HC group with a large effect size (Cohen's *d* = 1.727). ΔdBP at 1 min after standing in the OH (–) iRBD and OH (+) iRBD groups were significantly lower than that in the HC group with large effect sizes (Cohen's *d* = 1.057, 2.182, respectively).

Only dizziness was reported in this test as a subjective symptom in the OH (+) iRBD group (*n* = 2; 22.2%), and the incidence of dizziness was significantly different in the three groups (*p* = 0.002).

### Heart Rate Variability Analysis

**Figure 6:**
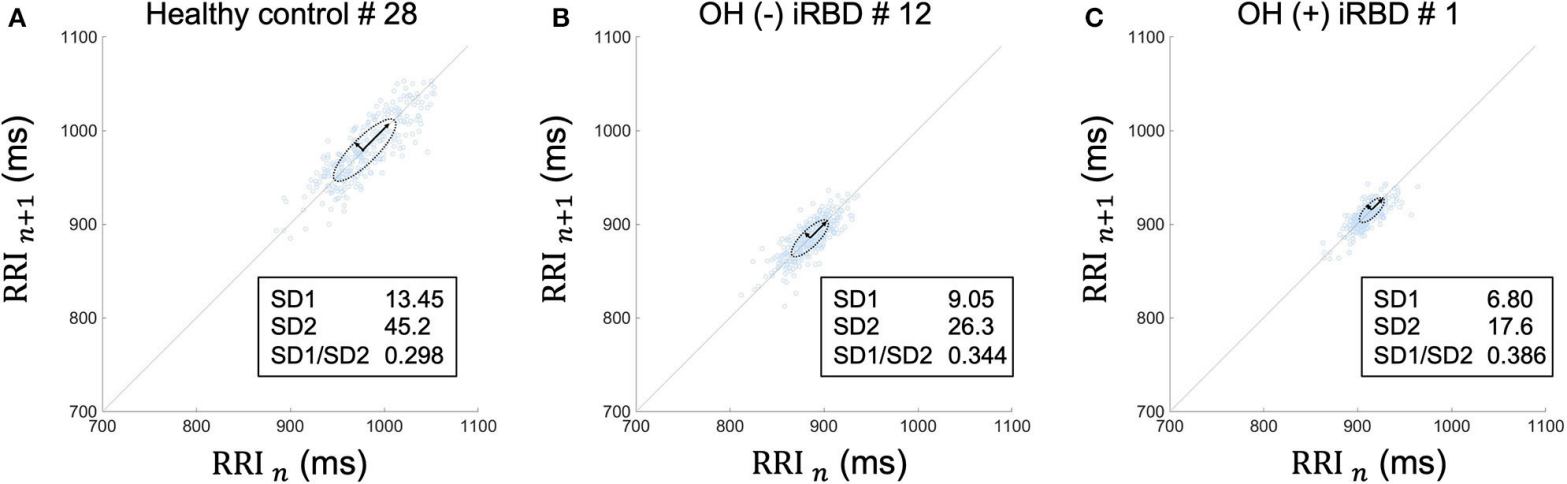
Poincaré plots during segment 2 in supine position of **(A)** a healthy control subject, **(B)** an OH (–) iRBD subject, and **(C)** an OH (+) iRBD subject. The Poincaré plot was based on a simple scatter of plots where the x value is the *n*th RRI and its corresponding y value is (*n* + 1)th RRI. **(A)** RRI plots were sparsely distributed between 900 and 1,050 ms. The distribution approximates a 45-degree tilted oval. **(B)** RRI plots were densely distributed between 840 and 920 ms. Both SD1 and SD2 were smaller than **(A)**. **(C)** RRI plots were closely distributed between 890 and 940 ms. SD1 and SD2 were smaller than **(A)**, in particular, SD2 was only ~40% of **(A)**. OH, orthostatic hypotension; iRBD, idiopathic rapid eye movement sleep behavior disorder; RRI, R–R intervals; HR, heart rate; SD1, standard deviation 1; SD2, standard deviation 2.

The results of the time-domain analysis and the Poincaré plots for HRV during segment 2 are shown in Table 3, and those during segment 1 and whole period are shown in Supplementary Table 1. Examples of Poincaré plots during segment 2 in the supine position are shown for a single HC subject, a single OH (–) iRBD subject, and a single OH (+) iRBD subject in Figure 6. HRV indices were significantly different among the three groups in time-domain analysis and the Poincaré plots.

**Table 3:** Results of heart rate variability analysis during segment 2 and comparison among healthy controls, OH (–) iRBD, and OH (+) iRBD groups.

						**Effect sizes (*****r*****)**		
	**HC**	**OH (–) iRBD**	**OH (+) iRBD**			**HC–**	**HC–**	**OH (–) iRBD–**
	**(*n* = 42)**	**(*n* = 12)**	**(*n* = 9)**	***p***	**Pairwise differences**	**OH (–) iRBD**	**OH (+) iRBD**	**OH (+) iRBD**
**Time-domain analysis**								
SDNN (ms)	34.9 (20.5)	21.0 (11.8)	13.8 (10.7)	**<0.001**	HC > OH (–) iRBD = OH (+) iRBD	**0.477**	**0.597**	0.380
RMSSD (ms)	19.9 (12.7)	13.9 (3.7)	9.5 (11.8)	**0.003**	HC > OH (–) iRBD = OH (+) iRBD	**0.364**	**0.358**	0.132
pNN50 (%)	1.14 (3.78)	0.00 (0.97)	0.60 (0.71)	**0.010**	HC > OH (–) iRBD	**0.370**	0.257	0.065
**Poincaré plots**								
SD1 (ms)	13.58 (6.44)	9.53 (3.53)	6.76 (8.32)	**0.003**	HC > OH (–) iRBD = OH (+) iRBD	**0.367**	**0.351**	0.588
SD2 (ms)	46.9 (30.4)	27.4 (16.6)	17.6 (12.8)	**<0.001**	HC > OH (–) iRBD = OH (+) iRBD	**0.471**	**0.602**	0.349
SD1/SD2	0.287 (0.112)	0.300 (0.254)	0.463 (0.172)	0.071	N/A	0.132	0.318	0.178

*HC, healthy control; iRBD, idiopathic rapid eye movement sleep behavior disorder; OH, orthostatic hypotension; SDNN, standard deviation of all N–N intervals; RMSSD, root mean square of successive differences; pNN50, percentage of successive RR intervals that differ by more than 50 ms; SD1, standard deviation 1; SD2, standard deviation 2*.

*Results are presented with median (interquartile range)*.

*Kruskal-Wallis test was conducted and post-hoc Mann-Whitney test followed by Bonferroni correction were used*.

*p < 0.05 was considered significant. Significant values are given in bold*.

*Effect sizes (r) are shown for pairwise difference*.

First, as for the time-domain analysis during segment 2, significant differences in SDNN, RMSSD, and pNN50 were confirmed among the three groups (*p* < 0.001, *p = 0.003*, 0, 010, *respectively*). Subsequent *post-hoc* analyses revealed that SDNN in the OH (–) iRBD and the OH (+) iRBD groups were significantly smaller than that in the HC group with medium and large effect sizes (*r* = 0.477, 0.597, *respectively*); RMSSD in the OH (–) iRBD and the OH (+) iRBD groups were significantly smaller than that in the HC group with medium effect sizes (*r* = 0.364, 0.358, *respectively*); and pNN50 in the OH (–) iRBD group was significantly smaller than that in the HC group with a medium effect size (*r* = 0.370).

Second, in terms of the Poincaré plots, there were no significant differences among the three groups in SD1/SD2; however, significant differences were confirmed in SD1 and SD2 (*p* = 0.003, *p* < 0.001, *respectively*). Subsequent *post-hoc* analyses revealed that SD1 in the OH (–) iRBD and the OH (+) iRBD groups were significantly smaller than that in the HC group with medium effect sizes (*r* = 0.367, 0.541, *respectively*); SD2 in the OH (–) iRBD and the OH (+) iRBD groups were significantly smaller than that in the HC group with medium and large effect sizes (*r* = 0.471, 0.602, *respectively*).

RRI usually fluctuates and the magnitude of the RRI fluctuations is represented by SDNN and RMSSD, or visualized by the Poincaré plot (Figure 3). The results in Table 3 are visualized in the examples in Figure 6. In the HC group, RRI fluctuated within a certain range, and the Poincaré plot showed an oval-like shape (Figure 6A). However, in the OH (–) iRBD group, RRI fluctuated within a small range, and the Poincaré plot showed a narrow distribution (Figure 6B) with a smaller median of SD1 and SD2 than those of the HC group (Table 3). In the OH (+) iRBD group, the Poincaré plot showed a narrow distribution as well as the OH (–) iRBD group (Figure 6C); in addition, the median of SD1 and SD2 were much smaller than those of the HC group (Table 3).

These results indicated that changes in HRV due to autonomic dysfunction may appear even in the supine position before standing, and iRBD patients, especially OH (+) iRBD patients, had smaller values of HRV indices than the HC group.

### Relationship Between OH (+/–) and HRV Indices

**Figure 7:**
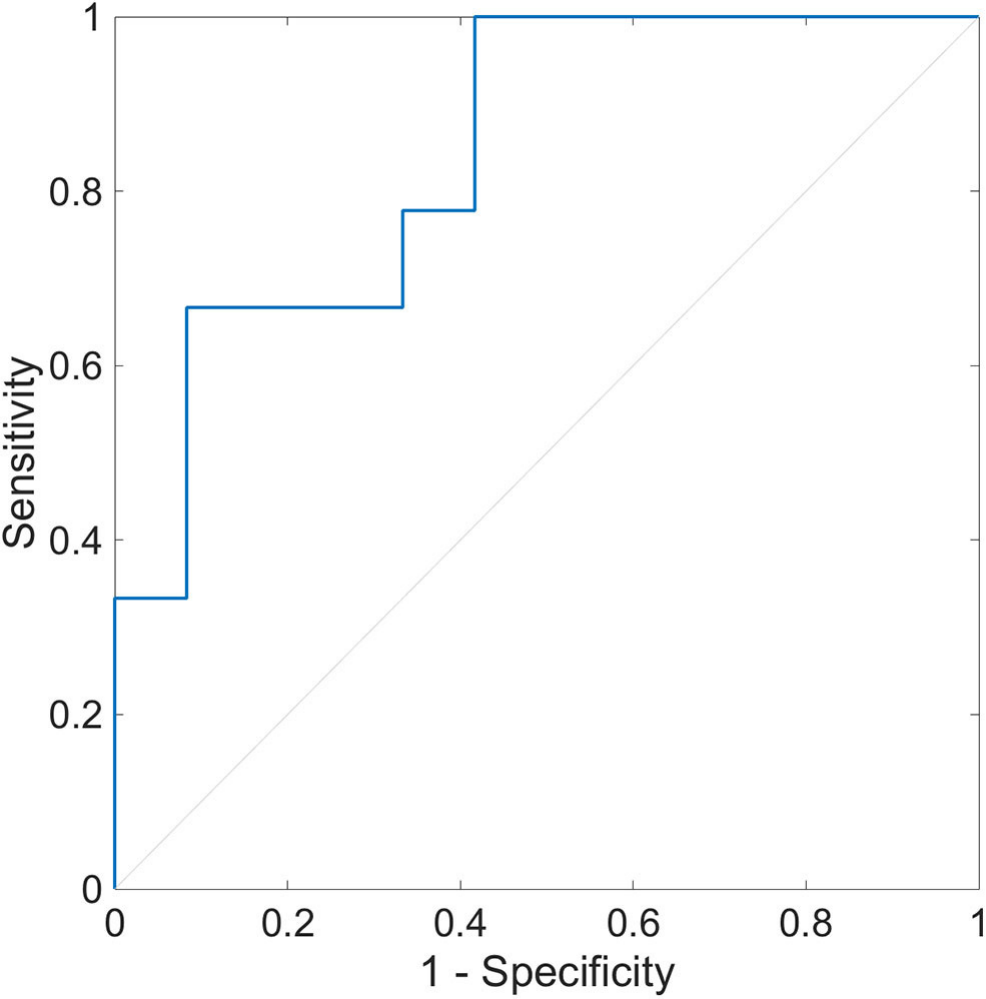
Receiver operating characteristic curve of heart rate variability during segment 2 for the detection of orthostatic hypotension. The ROC curve with the AUC of 0.840 is shown (*p* = 0.012). The sensitivity to OH (+) is 1.000, and the specificity to OH (–) is 0.583. The optimal cutoff point is pNN50 of 0.69 and SD2 of 17.6. ROC, receiver operating characteristic; AUC, area under the curve; OH (+), orthostatic hypotension-positive; OH (–), orthostatic hypotension-negative; pNN50, percentage of successive RR intervals that differ by more than 50 ms; SD2, standard deviation 2.

The multiple logistic regression analysis for OH (+/–) was performed using HRV indices during segment 2. We identified SD2 and pNN50 associated with OH (+/–) with the minimal value of BIC (*p* = 0.012). In this model, the AUC of the ROC curve was 0.840, and the sensitivity of OH (+) was 1.000, and the specificity of OH (–) was 0.583 (Figure 7). The cutoff values of SD2 and pNN50 were 17.6 and 0.69, respectively.

The same procedures were conducted for segment 1 and the whole period. SD2 and pNN50 were also associated with OH (+/–) with the minimal values of BIC in segment 1 and the whole period (*p* = 0.008, 0.005, *respectively*). In the segment 1 model (Supplementary Figure 1), the AUC of the ROC curve was 0.870, the sensitivity of OH (+) was 0.778, and the specificity of OH (–) was 0.917. The cutoff values of SD2 and pNN50 were 12.9 and 0.00, respectively. In the whole period model (Supplementary Figure 2), the AUC of the ROC curve was 0.870, the sensitivity of OH (+) was 0.889, and the specificity of OH (–) was 0.750. The cutoff values of SD2 and pNN50 were 21.0 and 0.12, respectively.

## Discussion

Orthostatic hypotension is a prominent symptom not only in neurodegenerative diseases such as PD, DLB, and MSA, but also in iRBD. It is important to find indicators of autonomic nerve activities associated with OH in order to prevent unintended falls. Dahms et al. conducted the orthostatic challenge test on patients with iRBD and healthy subjects (43), and they reported that 20 patients with iRBD showed significantly greater decrease in both sBP and dBP after standing than healthy controls; however, the decrease rate thereof was not clear. Miyamoto et al. reported that cardiac ^123^I-MIBG uptake decreased in patients with iRBD, which indicates cardiac sympathetic denervation (27, 28). In addition, the HRV data of iRBD patients were different from those of HC (30, 31). Thus, we aimed to investigate connections between rapid changes in HRV in the supine position and the OH occurrence.

Conventional time-domain and frequency-domain analyses have been used for HRV analysis in previous studies. Frequency-domain analysis, in particular, has been adopted for diagnosing the severity of autonomic dysfunction; however, the use of frequency-domain analysis is not appropriate for older people because frequency-domain analysis is significantly affected by arrhythmia and most older people have PVC and PAC (57, 58). In addition, it is difficult for conventional frequency-domain analysis to capture rapid changes in the autonomous nerve activities since it usually requires 2–5 min of RRI data. In order to deal with these problems, we adopted a robust HRV analysis method: a time-domain analysis and Poincaré plots for short-term HRV analysis after adopting the Hampel filter and the quotient filter for modifying arrhythmia appropriately.

Previous studies about the relationship between iRBD and the autonomic dysfunction mainly focused on nocturnal HRV data collected from PSG; however, there are some reports about HRV analysis using the RRI data collected during daytime. Rocchi et al. and Dahms found autonomic dysfunction in iRBD patients by analyzing HRV data collected during postural changes (43, 70). We also performed the orthostatic challenge test between 2 p.m. and 4 p.m. to investigate daytime changes in autonomic nerve activities.

It has been reported that HRV during rest is related to the severity of autonomic dysfunction. Diabetic autonomic dysfunction is related to increases in heart rate and RRI variations in the rest condition (71, 72). These indicate that HRV during rest, as well as during postural changes, may be useful for evaluating autonomic dysfunction. In addition, Sannino et al. tried to predict decreases in BP of young, healthy people after standing (56).

Our experiments revealed two important findings regarding HRV. The first was that short term HRV before standing was found to represent autonomic dysfunction in patients with iRBD. SDNN, RMSSD, pNN50, SD1, and SD2 differed in comparison in the three groups (Table 3). It is noteworthy that the HRV indices of iRBD patients in the supine position as well as in the standing position were different from those of the HC group, which is a new finding of this study. These results show that physiological RRI fluctuations are reduced in both of OH (–) and OH (+) iRBD patients (Figure 6), indicating sympathetic and the parasympathetic nervous dysfunction. In particular, SDNN and SD2 significantly differed from the OH (+) iRBD and HC groups with large effect sizes, thus OH (+) iRBD patients were indicated to have severe autonomic dysfunction compared to HC. Decreases in HRV indices in OH (–) and OH (+) iRBD are in good agreement with previous studies (30, 31).

The second finding is that the combination of HRV indices showed the possibility of predicting OH. Multivariate logistic regression analysis for OH (–) and OH (+) iRBD patients using HRV indices presented a good model and ROC curve (Figure 7). HRV indices may reflect the severity of autonomic dysfunction in iRBD patients, which may have led to the relationship between HRV indices and OH. These findings were obtained by the analysis of HRV during segment 2; similar results were obtained by the analysis of HRV during segment 1 and the whole period (Supplementary Table 1, Supplementary Figures 1, 2).

Our results are of clinical importance in terms of showing the possibility that OH of older people can be predicted using only HRV data in the supine position without an orthostatic challenge test, which could improve the efficiency and safety of testing. However, several steps are required for the clinical application of OH detection by HRV indices. Since the number of OH patients were limited in this study, we performed a multivariate logistic regression analysis for only the construction of models. In the future, it is needed to validate the current model. Besides there are issues to be addressed to meet the clinical needs, such as whether using only the HRV indices is enough for OH detection or whether a combination of the OH questionnaire and the HRV indices is useful. A new device that can predict the occurrence of a fall can thereby be realized, since a wearable device for measuring RRI and calculating HRV in real-time has already been developed, which would contribute to preventing the falls of older people.

There were two benefits of recruiting iRBD patients in this study. The first was that the evaluation of autonomic dysfunction in the whole population of iRBD patients [OH (–) and OH (+) iRBD] was carried out, and the second was that we were able to perform an analysis to detect severe cases of autonomic dysfunction [OH (+) iRBD group]. iRBD patients have a high rate of autonomic dysfunction, and the degree of autonomic dysfunction was considered to be graded from mild to severe. Since PD, DLB, and MSA are associated with severe autonomic dysfunction, and most of the iRBD patients phenoconvert to PD, DLB, and MSA over a long period of time (14, 15, 20), patients with iRBD were thought to have various degrees of autonomic dysfunction. In fact, in this study, iRBD patients were split approximately halfway between OH (–) and OH (+), and there were significant decreases in BP after standing and decreases in HRV indices during the supine position in the OH (–) and OH (+) iRBD groups compared to HC (Tables 2, 3). Therefore, the recruitment of iRBD patients, who were diagnosed with autonomic dysfunction and had had its severity graded, was consistent with the purpose of this study.

Demographics of the patients with iRBD included in our experiments were not much different from previous studies (73), although previous reports on OH in iRBD patients vary widely in their testing methods and results. Lee reported that 59% (10 of 17) of patients with iRBD displayed OH during the tilt test, and it was confirmed that 94% of patients had some autonomic dysfunction through a composite autonomic severity score which comprehensively evaluates autonomic nervous dysfunction (74). In Fraucher's study, only 13% (2 of 15) of patients with iRBD had OH; however, the composite autonomic scoring scale was significantly higher in patients with iRBD than healthy controls (75). A recent multicenter study found that 29% (156 of 531) of patients with iRBD had orthostatic symptoms (26). Although these studies have reported that most patients with iRBD have autonomic dysfunction, the proportion of OH in iRBD patients in these studies was different from each other. This variation might be explained by the age of the participants, duration of iRBD, the prevalence of cardiovascular disease, and experimental techniques. On the other hand, 43% (9 of 21) of patients with iRBD displayed OH (Tables 1, 2) in this study, which is a modest prevalence in comparison with these previous studies. Patients with iRBD is prevalent in older males (76), and the population of this study matched the epidemiological characteristics of iRBD. In addition, patients with dementia were excluded, and we confirmed that their MMSE scores exceeded the cutoff. It is also consistent with previous reports, in which only iRBD patients with no apparent cognitive impairment were included (73). Thus, it is concluded that our study has external validity.

On the other hand, the RBDSQ-J score of our population was lower than in the previous report (53), and the average disease duration was rather long: ~10 years after symptom emergence. The RBDSQ-J score may have been low due to the long disease duration of iRBD (77). According to a longitudinal observational study of patients with a long disease duration of iRBD, almost all subjects showed multiple features of neurodegeneration including autonomic dysfunction after a follow-up of at least 10 years (19). Therefore, the participants in the present study with a long disease duration are an appropriate population for evaluating autonomic dysfunction.

The time of the experiment, the last meal, and the temperature and humidity in the laboratory were controlled to reduce their effects on the autonomic activities. The participants were instructed to stop consuming alcohol and tobacco the night before. However, autonomic activities in daily life are affected by various factors such as circadian rhythms and diet as well as alcohol, tobacco, and temperature (40, 42, 78–80). Niu et al. demonstrated significant changes in HRV after hemodialysis in elderly patients with diabetes (81). Thus, changes in the autonomic nervous activities, including HRV and BP fluctuations in daily life, may be greater than the results of this study. In order to accurately identify the indicators of OH occurrence, it is necessary to record the amount of daily alcohol and water consumption, the time of meal, temperature, humidity, and other factors as well as the RRI data. Since our device can measure RRI continuously for 24 h, we will perform additional experiments in a daily-life setting.

Because Xie et al. suggested that cognitive impairment-related gait disorders occur while walking in daily life among patients with amnestic mild cognitive impairment (82), comprehensive assessments of falls including the autonomic nervous functions and cognitive impairment-related gait disorders are also needed. In addition, from the viewpoint of fall prevention, we should account for orthostatic cerebral hypoperfusion syndromes, which are possible underlying pathophysiologies of orthostatic dizziness without orthostatic hypotension (83).

The limitations of this research include medication and comorbidities; although older people regularly use blood pressure-related medications due to hypertension or complications of angina, our study did not control for these medications and comorbidities. Some patients took calcium channel blockers, ACE and AT II inhibitors, and organic nitrates. There were significant differences in the proportion of myocardial infarction comorbidity and the use of ACE and AT II inhibitors (Table 1). To address this problem, we performed an analysis related to HRV after excluding patients with the comorbidity of myocardial infarction or the use of ACE and AT II inhibitors. After excluding one OH (–) iRBD patient and two OH (+) iRBD patients, HRV indices were calculated and the multivariate logistic regression analysis was performed (Supplementary Table 2, Supplementary Figure 3). The results of the analysis showed similar results in Table 3 and Figure 7. Although the patients with other comorbidities or medication use should have been excluded from the analysis, we could not perform the analysis due to the reduced sample size. The undeniable influence of some comorbidities and medication is a limitation of this study. In order to assess the autonomic dysfunction without drug-related effects, it was necessary to instruct the subjects to discontinue these medications. Dahms performed the orthostatic challenge test after having the patients stop taking their regular medications (43); however, it would not have been appropriate for the patients to stop taking their regular medication for our experiments, because our purpose was to identify indicators of risk of falls in daily life.

Another limitation was the ratio of male to female in the HC group. In this study, there were more females than males in HC (female: 62%), although the number of males was more significant than that of females in the patient group reflecting the characteristics of RBD prevalence. There was the possibility that this gender difference might affect HRV and decreases in BP. To address this issue, we prepared randomized HC samples with the number of HC adjusted for the ratio of males to females in the iRBD groups (female: 19%) because the sample size of iRBD was smaller than that of HC. Namely, of the 26 HC females, we randomly selected 4 females 10 times, then created a sample of HC (16 males and 4 females) 10 times. The mean or median of variables of the 10 randomized samples of the 20 HC, whose male-to-female ratio was matched to the iRBD, are shown in Supplementary Table 3. Although the comorbidities with arterial hypertension and diabetes mellitus and the rate of medication use of calcium channel blockers and ACE and AT II inhibitors were higher than the original HC group (Table 1), the values of BP and PR and the HRV indices were not markedly different from those of the original HC group (Tables 2, 3). Therefore, we believe that the gender ratio in HC does not largely affect the results of this study.

## Conclusion

In this study, iRBD patients showed significant decreases in BP after standing and significant decreases in HRV indices during a supine position compared to HC. The combination of HRV indices would predict subsequent OH in iRBD patients. Our results are clinically useful in terms of showing the possibility that OH can be predicted without an orthostatic challenge test using only HRV data in the supine position, which could improve the efficiency and the safety of testing. Future studies are required to develop a system that can predict OH before standing up by analyzing HRV in real-time.

## Data Availability Statement

The datasets analyzed in the current study are available from the corresponding author upon reasonable request.

## Ethics Statement

The studies involving human participants were reviewed and approved by the ethical committee at Shiga University of Medical Science. The patients/participants provided their written informed consent to participate in this study.

## Author Contributions

YS, MM, HK, KF, and CN conceived the idea. YS, CN, and TK performed the experiments and analyzed the data. KF and TY contributed to the reagents, materials, and analysis tools. YS, KF, and HK wrote the draft. MM, YO, HK, KF, CN, YG, MK, TY, MH-O, and KO interpreted the data and revised the manuscript. All authors contributed to the article and approved the submitted version.

## Conflict of Interest

HK's laboratory is supported by a donation from Fukuda Lifetech Co., Ltd., Fukuda Life Tech Keiji Co., Ltd., Tanaka Sleep Clinic, Akita Sleep Clinic, and Ai Care Co., Ltd. to the Shiga University of Medical Science. HK received a grant from Merck Sharp & Dohme Corp/MSD K.K. (the Investigator-Initiated Studies Program). The opinions expressed in this paper are those of the authors and do not necessarily represent those of Merck Sharp & Dohme Corp/MSD K.K. KF, TY, and MK participate in Quadlytics Inc. The remaining authors declare that the research was conducted in the absence of any commercial or financial relationships that could be construed as a potential conflict of interest.

## Supplementary Material

Supplementary Figure 1 Receiver operating characteristic curve of heart rate variability during segment 1 for the detection of orthostatic hypotension. The ROC curve with the AUC of 0.870 is shown (*p* = 0.008). The sensitivity to OH (+) is 0.778, and the specificity to OH (–) is 0.917. The optimal cutoff point is pNN50 of 0.00 and SD2 of 12.9. ROC, receiver operating characteristic; AUC, area under the curve; OH (+), orthostatic hypotension-positive; OH (–), orthostatic hypotension-negative; pNN50, percentage of successive RR intervals that differ by more than 50 ms; SD2, standard deviation 2.

Supplementary Figure 2 Receiver operating characteristic curve of heart rate variability during the whole period for the detection of orthostatic hypotension. The ROC curve with the AUC of 0.870 is shown (*p* = 0.005). The sensitivity to OH (+) is 0.889, and the specificity to OH (–) is 0.750. The optimal cutoff point is pNN50 of 0.12 and SD2 of 21.0. ROC, receiver operating characteristic; AUC, area under the curve; OH (+), orthostatic hypotension-positive; OH (–), orthostatic hypotension-negative; pNN50, percentage of successive RR intervals that differ by more than 50 ms; SD2, standard deviation 2.

Supplementary Figure 3 Receiver operating characteristic curve of heart rate variability during segment 2 for the detection of orthostatic hypotension, excluding the patients with a history of myocardial infarction or use of angiotensin-converting-enzyme or angiotensin II type 1 inhibitors. The ROC curve with the AUC of 0.818 is shown (*p* = 0.023). The sensitivity to OH (+) is 1.000, and the specificity to OH (–) is 0.636. The optimal cutoff point is pNN50 of 0.71 and SD2 of 23.9. ROC, receiver operating characteristic; AUC, area under the curve; OH (+), orthostatic hypotension-positive; OH (–), orthostatic hypotension-negative; pNN50, percentage of successive RR intervals that differ by more than 50 ms; SD2, standard deviation 2.

Supplementary Table 1 Results of heart rate variability analysis during segment 1 and the whole period and comparison among healthy controls, OH (–) iRBD, and OH (+) iRBD groups.

Supplementary Table 2 Results of heart rate variability analysis during segment 2 of OH (–) iRBD and OH (+) iRBD groups without history of myocardial infarction or use of angiotensin-converting-enzyme or angiotensin II type 1 inhibitors.

Supplementary Table 3 Demographic data and results of the study of randomly sampled, sex ratio-adjusted healthy control.
