# Supplementary figures and images for: Resting Heart Rate Variability Is Associated With Subsequent Orthostatic Hypotension: Comparison Between Healthy Older People and Patients With Rapid Eye Movement Sleep Behavior Disorder
Source: Front Neurol. 2020 Nov 23;11:567984. doi: 10.3389/fneur.2020.567984 (PMC7719719; doi:10.3389/fneur.2020.567984)

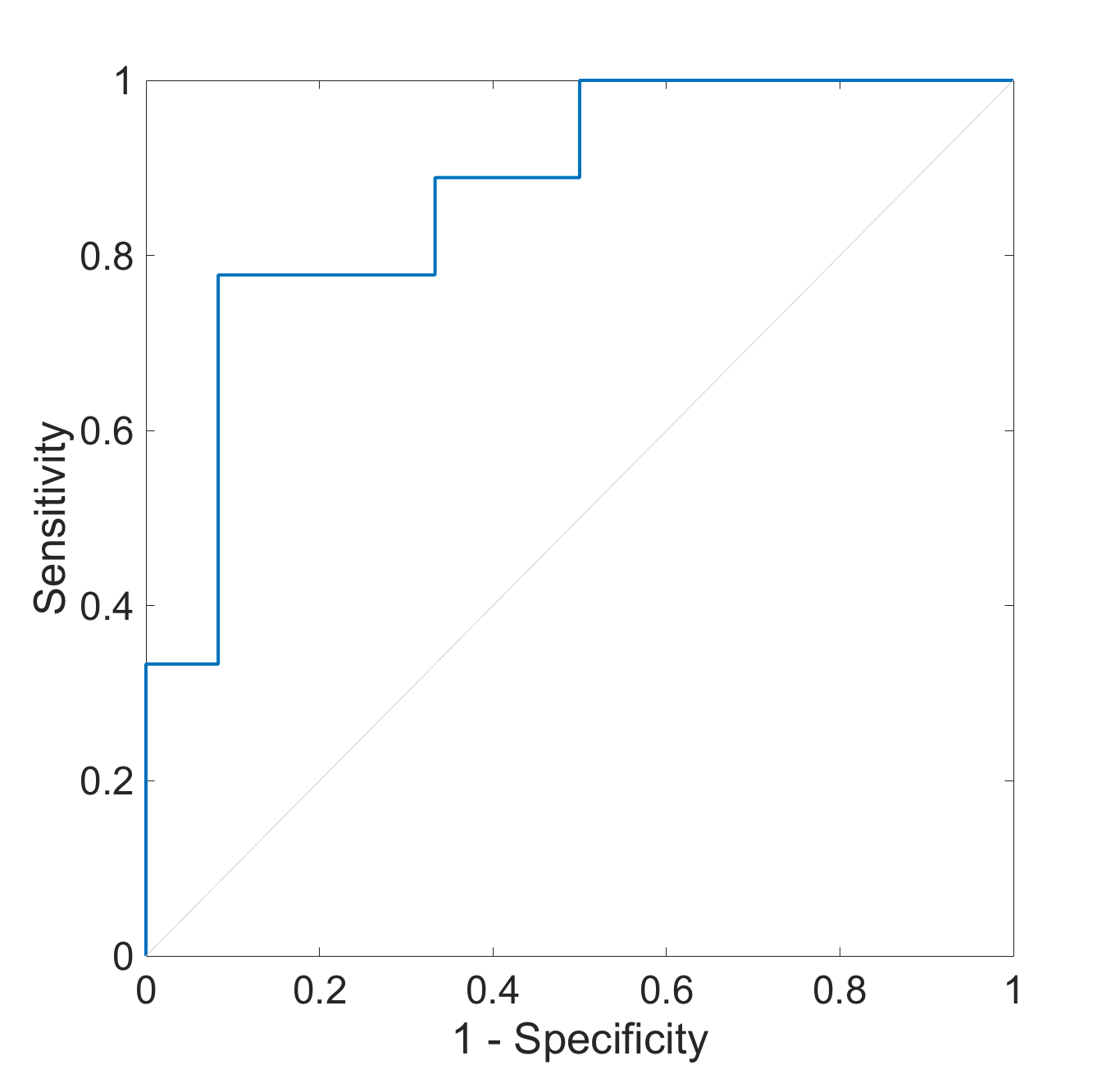

Supplement: Supplementary Figure 1 — Receiver operating characteristic curve of heart rate variability during segment 1 for the detection of orthostatic hypotension. The ROC curve with the AUC of 0.870 is shown (p = 0.008). The sensitivity to OH (+) is 0.778, and the specificity to OH (–) is 0.917. The optimal cutoff point is pNN50 of 0.00 and SD2 of 12.9. ROC, receiver operating characteristic; AUC, area under the curve; OH (+), orthostatic hypotension-positive; OH (–), orthostatic hypotension-negative; pNN50, percentage of successive RR intervals that differ by more than 50 ms; SD2, standard deviation 2. [file Image_1.TIFF]

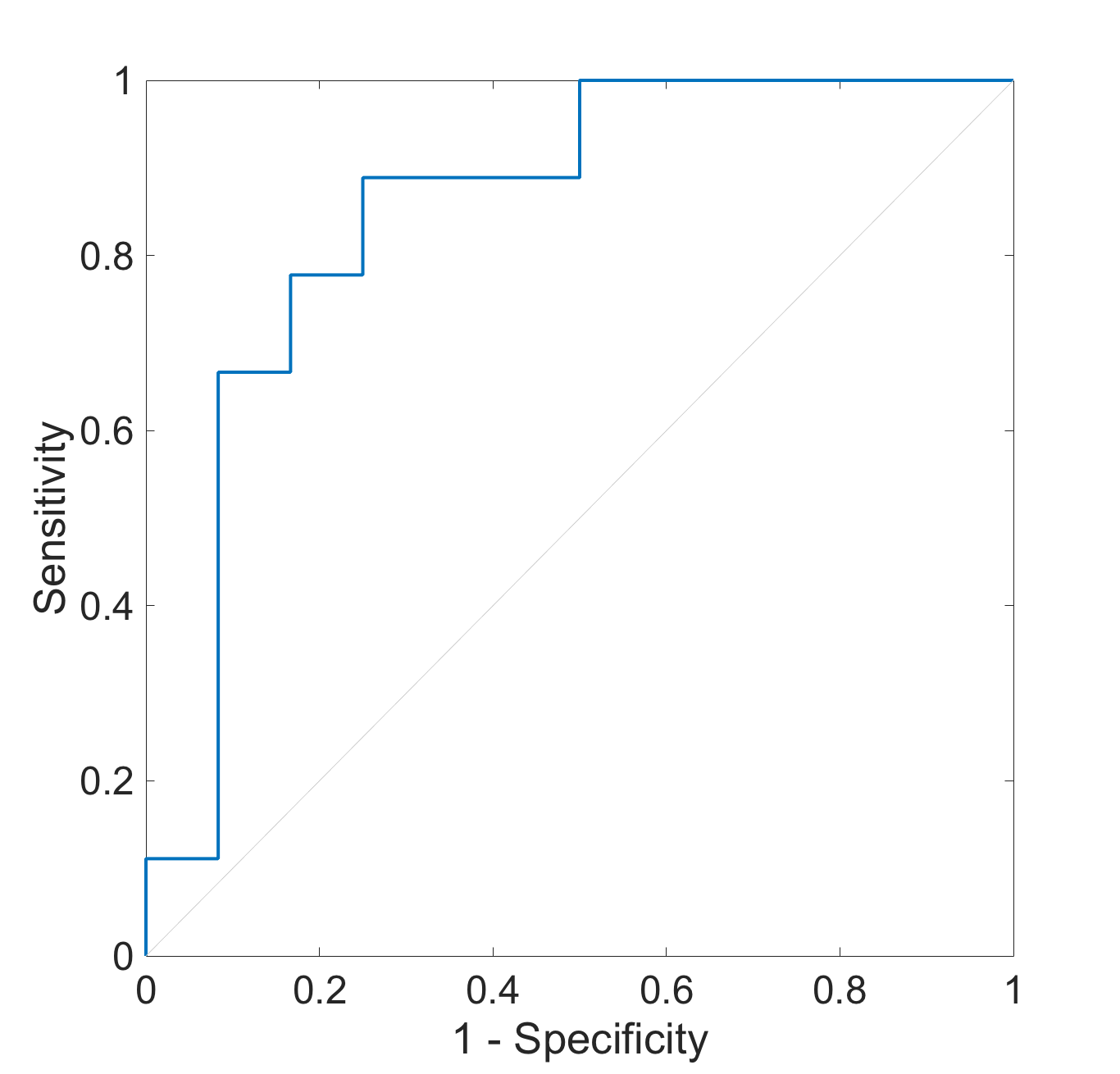

Supplement: Supplementary Figure 2 — Receiver operating characteristic curve of heart rate variability during the whole period for the detection of orthostatic hypotension. The ROC curve with the AUC of 0.870 is shown (p = 0.005). The sensitivity to OH (+) is 0.889, and the specificity to OH (–) is 0.750. The optimal cutoff point is pNN50 of 0.12 and SD2 of 21.0. ROC, receiver operating characteristic; AUC, area under the curve; OH (+), orthostatic hypotension-positive; OH (–), orthostatic hypotension-negative; pNN50, percentage of successive RR intervals that differ by more than 50 ms; SD2, standard deviation 2. [file Image_2.TIFF]

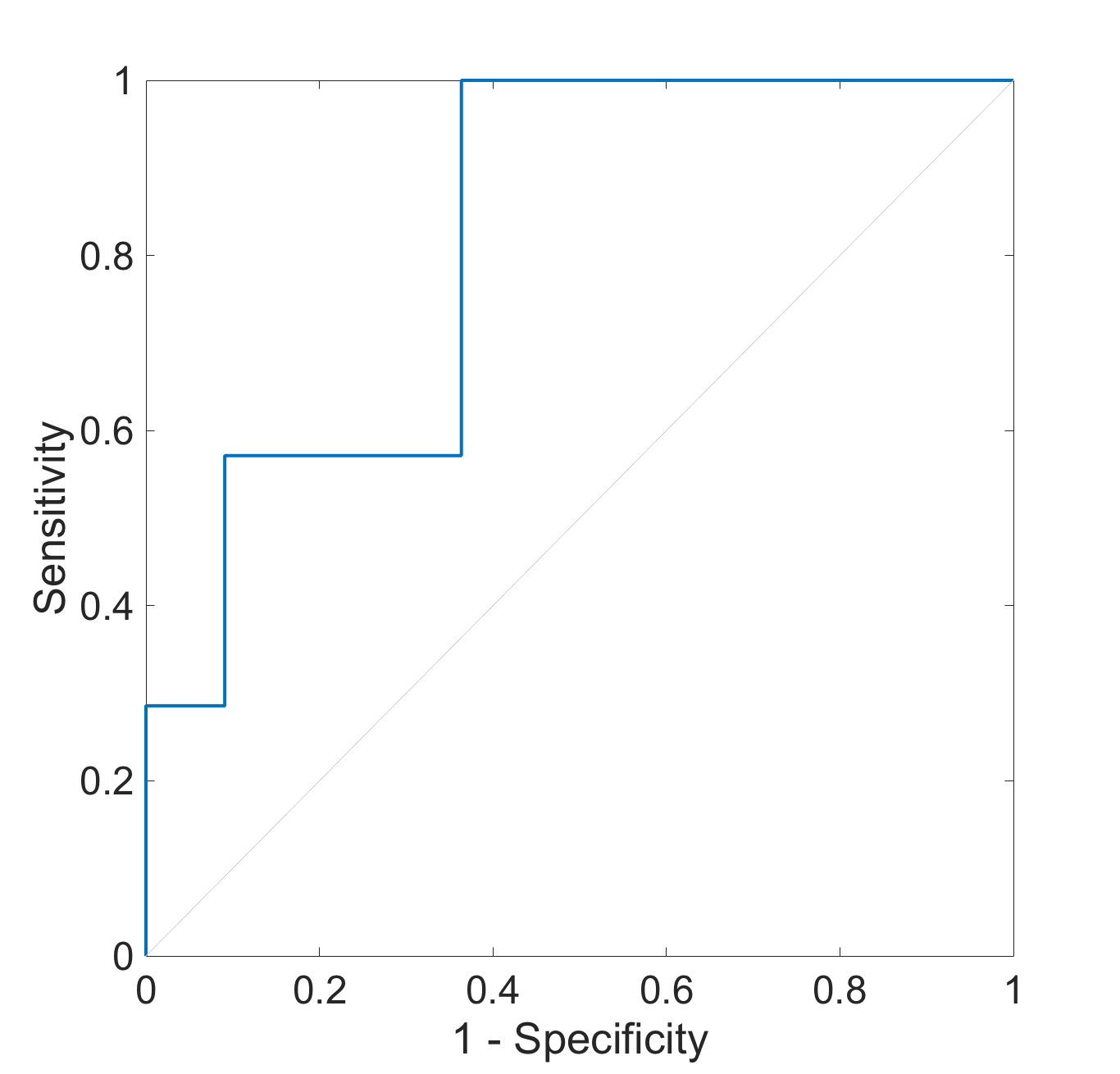

Supplement: Supplementary Figure 3 — Receiver operating characteristic curve of heart rate variability during segment 2 for the detection of orthostatic hypotension, excluding the patients with a history of myocardial infarction or use of angiotensin-converting-enzyme or angiotensin II type 1 inhibitors. The ROC curve with the AUC of 0.818 is shown (p = 0.023). The sensitivity to OH (+) is 1.000, and the specificity to OH (–) is 0.636. The optimal cutoff point is pNN50 of 0.71 and SD2 of 23.9. ROC, receiver operating characteristic; AUC, area under the curve; OH (+), orthostatic hypotension-positive; OH (–), orthostatic hypotension-negative; pNN50, percentage of successive RR intervals that differ by more than 50 ms; SD2, standard deviation 2. [file Image_3.TIFF]
