# Supplementary material for: Resting Heart Rate Variability Is Associated With Subsequent Orthostatic Hypotension: Comparison Between Healthy Older People and Patients With Rapid Eye Movement Sleep Behavior Disorder
Source: Front Neurol. 2020 Nov 23;11:567984. doi: 10.3389/fneur.2020.567984 (PMC7719719; doi:10.3389/fneur.2020.567984)
Supplement: Supplementary Table 1 — Results of heart rate variability analysis during segment 1 and the whole period and comparison among healthy controls, OH (–) iRBD, and OH (+) iRBD groups. [file Table_1.pdf]

Supplementary Table 1
Results of heart rate variability analysis during segment 1 and whole period and comparison among healthy controls, OH (-) iRBD, and OH (+) iRBD groups.

						Effect sizes (*r*)		
	HC (n = 42)	OH (-) iRBD (n = 12)	OH (+) iRBD (n = 9)	p	Pairwise differences	HC - OH (-) iRBD	HC - OH (+) iRBD	OH (-) iRBD - OH (+) iRBD
**segment 1**								
**Time-domain analysis**								
SDNN (ms)	30.7 (15.3)	19.0 (10.0)	12.2 (8.4)	**< 0.001**	HC > OH (-) iRBD = OH (+) iRBD	**0.446**	**0.507**	0.240
RMSSD (ms)	19.0 (11.8)	12.9 (3.2)	8.5 (13.3)	**0.005**	HC > OH (-) iRBD	**0.355**	0.333	0.132
pNN50 (%)	1.01 (4.83)	0.00 (0.72)	0.00 (1.08)	**0.024**	HC > OH (-) iRBD	**0.344**	0.209	0.069
**Poincaré plots**								
SD1 (ms)	13.11 (6.84)	8.96 (3.37)	5.99 (8.95)	**0.004**	HC > OH (-) iRBD = OH (+) iRBD	**0.358**	**0.337**	0.116
SD2 (ms)	39.9 (20.9)	25.5 (13.2)	15.5 (9.0)	**<0.001**	HC > OH (-) iRBD = OH (+) iRBD	**0.453**	**0.517**	0.287
SD1/SD2	0.303 (0.088)	0.340 (0.211)	0.400 (0.320)	0.410	N/A	0.079	0.178	0.132
**whole period**								
**Time-domain analysis**								
SDNN (ms)	34.3 (16.0)	21.1 (10.1)	15.1 (9.3)	**< 0.001**	HC > OH (-) iRBD = OH (+) iRBD	**0.471**	**0.566**	0.349
RMSSD (ms)	19.7 (10.6)	13.9 (4.0)	9.0 (12.7)	**0.003**	HC > OH (-) iRBD = OH (+) iRBD	**0.372**	**0.358**	0.147
pNN50 (%)	1.04 (4.30)	0.15 (0.77)	0.29 (0.89)	**0.004**	HC > OH (-) iRBD	**0.407**	0.277	0.096

**Poincaré plots**								
SD1 (ms)	13.70 (7.10)	9.78 (3.06)	6.38 (8.61)	**0.002**	HC > OH (-) iRBD = OH (+) iRBD	**0.375**	**0.358**	0.122
SD2 (ms)	45.3 (22.8)	27.8 (13.3)	21.0 (10.2)	**<0.001**	HC > OH (-) iRBD = OH (+) iRBD	**0.460**	**0.580**	0.302
SD1/SD2	0.273 (0.093)	0.294 (0.220)	0.393(0.304)	0.349	N/A	0.081	0.194	0.163

HC, healthy control; iRBD, idiopathic rapid eye movement sleep behavior disorder; OH, orthostatic hypotension

SDNN, standard deviation of all N–N intervals;

RMSSD, root mean square of successive differences;

pNN50, percentage of successive RR intervals that differ by more than 50 ms;

SD1, standard deviation 1;

SD2, standard deviation 2;

Results are presented with median (interquartile range).

Kruskal-Wallis test was conducted and post hoc Mann-Whitney test followed by Bonferroni correction were used.

$p < 0.05$ was considered significant. Significant values are given in bold.

Effect sizes (*r*) are shown for pairwise difference

Supplementary Table 2

Results of heart rate variability analysis during segment 2 of OH (-) iRBD and OH (+) iRBD groups wihtout history of myocardial infarction or use of angiotensin-converting-enzyme or angiotensin II type 1 inhibitors

	OH (-) iRBD (n = 11)	OH (+) iRBD (n = 7)
**Time-domain analysis**		
SDNN (ms)	22.4 (12.1)	14.0 (8.1)
RMSSD (ms)	13.6 (4.2)	11.7 (11.8)
pNN50 (%)	0.00 (1.00)	0.69 (0.67)
**Poincaré plots**		
SD1 (ms)	9.05 (4.10)	6.80 (8.68)
SD2 (ms)	28.5 (17.7)	19.6 (10.1)
SD1/SD2	0.257 (0.174)	0.553 (0.283)

iRBD, idiopathic rapid eye movement sleep behavior disorder; OH, orthostatic hypotension

SDNN, standard deviation of all N–N intervals;

RMSSD, root mean square of successive differences;

pNN50, percentage of successive RR intervals that differ by more than 50 ms;

SD1, standard deviation 1; SD2, standard deviation 2;

Results are presented with median (interquartile range).

Supplementary Table 3

Demographic data and results of the study of randomly sampled, sex ratio-adjusted healthy control.

	Randomly sampled, sex ratio-adjusted healthy control (n = 20: male 16, female 4)
Age (years)	69.2 ± 5.0
BMI (kg/m^2)	23.3 ± 2.9
Years of education	13.0 (4.0)
MMSE	29.0 (4.0)
RBDSQ-J	1.0 (3.0)
Comorbidities	
Arterial hypertension (n)	6.1 (30.5%)
Coronary artery disease (n)	0.2 (1.0%)
Myocardial infarction (n)	0.0 (0.0%)
Diabetes mellitus (n)	2.2 (11.0%)
Medication	
Calcium channel blockers (n)	5.2 (26.0%)
ACE and AT II inhibitors (n)	4.0 (20.0%)
Organic nitrates (n)	0.2 (1.0%)
Systolic BP (mm Hg)	
Baseline	134.6 ± 16.6
ΔsBP at 1 minute after standing	-3.4 ± 10.4
ΔsBP at 3 minutes after standing	1.1 ± 10.3
Diastolic BP (mm Hg)	
Baseline	80.2 ± 7.7
ΔdBP at 1 minute after standing	1.2 ± 6.5
ΔdBP at 3 minutes after standing	4.6 ± 6.0
Pulse Rate (beats/min)	
Baseline	61.8 ± 7.3
ΔPR at 1 minute after standing	10.8 ± 5.4
ΔPR at 3 minutes after standing	7.7 ± 5.4
Subjective Symptoms	
dizziness	0 (0.0%)

**HRV indices during segment 2**	
**Time-domain analysis**	
SDNN (ms)	38.9 (18.7)
RMSSD (ms)	20.8 (14.9)
pNN50 (%)	1.41 (4.19)
**Poincaré plots**	
SD1 (ms)	14.73 (8.35)
SD2 (ms)	51.7 (25.3)
SD1/SD2	0.296 (0.129)

ACE, angiotensin-converting-enzyme; AT II, angiotensin II type 1; BMI, body mass index;
MMSE, Mini-Mental State Examination; RBDSQ-J, rapid eye movement sleep behavior disorder screening questionnaire Japanese version.

BP, blood pressure; PR, pulse rate.

SDNN, standard deviation of all N–N intervals;

RMSSD, root mean square of successive differences;

pNN50, percentage of successive RR intervals that differ by more than 50 ms;

SD1, standard deviation 1;

SD2, standard deviation 2;

The mean or median of the 10 samples of 20 HC subjects obtained by randomized sampling are shown.
Results are presented with mean ± standard deviation for parametric variables, median (interquartile range), or percentage in parentheses.
